# Machine Learning Prediction of Short Cervix in Mid-Pregnancy Based on Multimodal Data from the First-Trimester Screening Period: An Observational Study in a High-Risk Population

**DOI:** 10.3390/biomedicines13092057

**Published:** 2025-08-23

**Authors:** Shengyu Wu, Jiaqi Dong, Jifan Shi, Xiaoxian Qu, Yirong Bao, Xiaoyuan Mao, Mu Lv, Xuan Chen, Hao Ying

**Affiliations:** 1 Department of Obstetrics, Shanghai First Maternity and Infant Hospital, School of Medicine, Tongji University; Shanghai Key Laboratory of Maternal Fetal Medicine, Shanghai Institute of Maternal-Fetal Medicine and Gynecologic Oncology, Shanghai 200092, China; 2211568@tongji.edu.cn (S.W.); 1910866@tongji.edu.cn (J.D.); quxiaoxian@51mch.com (X.Q.); baoyirong@51mch.com (Y.B.); maoxiaoyuan@51mch.com (X.M.); 2311233@tongji.edu.cn (M.L.); 2332329@tongji.edu.cn (X.C.); 2 Research Institute of Intelligent Complex Systems, Fudan University, Shanghai 200433, China; jfshi@fudan.edu.cn; 3 State Key Laboratory of Medical Neurobiology and MOE Frontiers Center for Brain Science, Fudan University, Shanghai 200032, China; 4 Shanghai Artificial Intelligence Laboratory, Shanghai 200232, China

**Keywords:** short cervix, mid-pregnancy, prediction model, machine learning, preterm birth

## Abstract

**Background**: A short cervix in the second trimester significantly increases preterm birth risk, yet no reliable first-trimester prediction method exists. Current guidelines lack consensus on which women should undergo transvaginal ultrasound (TVUS) screening for cost-effective prevention. Therefore, it is vital to establish a highly accurate and economical method for use in the early stages of pregnancy to predict short cervix in mid-pregnancy. **Methods**: A total of 1480 pregnant women with singleton pregnancies and at least one risk factor for spontaneous preterm birth (<37 weeks) were recruited from January 2020 to December 2020 at the Shanghai First Maternity and Infant Hospital, Tongji University School of Medicine. Cervical length was assessed at 20–24 weeks of gestation, with a short cervix defined as <25 mm. Feature selection employed tree models, regularization, and recursive feature elimination (RFE). Seven machine learning models (logistic regression, linear discriminant analysis, k-nearest neighbors, support vector machine, decision tree, random forest, XGBoost) were trained to predict mid-trimester short cervix. The XGBoost model—an ensemble method leveraging sequential decision trees—was analyzed using Shapley Additive Explanation (SHAP) values to assess feature importance, revealing consistent associations between clinical predictors and outcomes that align with known clinical patterns. **Results**: Among 1480 participants, 376 (25.4%) developed mid-trimester short cervix. The XGBoost-based prediction model demonstrated high predictive performance in the training set (Recall = 0.838, F1 score = 0.848), test set (Recall = 0.850, F1 score = 0.910), and an independent dataset collected in January 2025 (Recall = 0.708, F1 score = 0.791), with SHAP analysis revealing pre-pregnancy BMI as the strongest predictor, followed by second-trimester pregnancy loss history, peripheral blood leukocyte count (WBC), and positive vaginal microbiological culture results (≥10^5^ CFU/mL, measured between 11^+0^ and 13^+6^ weeks). **Conclusions**: The XGBoost model accurately predicts mid-trimester short cervix using first-trimester clinical data, providing a 6-week window for targeted interventions before the 20–24-week gestational assessment. This early prediction could help guide timely preventive measures, potentially reducing the risk of spontaneous preterm birth (sPTB).

## 1. Introduction

Preterm birth (<37 weeks gestational age), particularly spontaneous preterm birth (sPTB), remains the leading cause of neonatal mortality and long-term complications such as neurological deficits [1]. One of the strongest predictors of sPTB is a short cervix, typically defined as a cervical length less than 25 millimeters (mm), measured by transvaginal ultrasound (TVUS) between 14 and 28 gestational weeks in the second trimester [2,3,4]. Evidence shows that for every 1 mm reduction in cervical length during mid-pregnancy, the risk of sPTB increases by approximately 3% [5], highlighting the importance of timely identification.

However, the short time window between mid-pregnancy and preterm birth makes early intervention for cervical shortening particularly challenging in clinical practice. Currently, there are no reliable early methods for predicting short cervix in mid-pregnancy, which limits timely and effective preventive measures. While TVUS remains the gold standard for cervical length assessment, there is no consensus on which populations should be screened. Universal screening—though more effective in reducing sPTB than selective or opt-in approaches—imposes significant economic burdens [6], and some studies have shown no significant difference in sPTB rates between women who universal TVUS and those who do not [7,8], raising concerns about the necessity and cost-effectiveness of universal screening. An alternative is risk factor–based screening, where TVUS is limited to women with one or more known risk factors. This strategy can reduce unnecessary TVUS examinations, but its diagnostic performance is suboptimal, with moderate sensitivity (60.4%) and specificity (62.8%). Narrowing the criteria to women with two or more independent risk factors greatly improves specificity (96.5%) but at the cost of drastically reduced sensitivity (14.6%), leading to a high rate of missed cases and undermining its clinical utility [9,10]. Furthermore, in real-world clinical settings, many high-risk women decline TVUS screening due to discomfort, cost, or personal concerns, further increasing the likelihood of underdiagnosis [11].

Therefore, it is essential, particularly for pregnant women with high-risk factors for sPTB, to develop a more cost-effective and simple approach for use in the early stages of pregnancy to predict short cervix in mid-pregnancy. This is also a crucial step in developing more effective prevention and treatment approaches. This type of approach can not only enable clinical doctors to take corresponding measures to prevent short cervix in mid-pregnancy as soon as possible but also provide the best balance for diagnostic cost-effectiveness.

At present, machine learning (ML) algorithms have been widely implemented to detect diseases and predict outcomes [12,13,14]. These indices are employed to predict preterm birth. For example, Tal Korem et al. used ML models to predict sPTB risk based on metabolite levels [15]. However, the role of cervical length changes in preterm birth is still somewhat neglected in these models. In addition, there are currently no ML models for predicting short cervix in mid-pregnancy. In this study, we sought to address this gap by examining whether routinely available clinical and laboratory data from the first trimester (11^+0^ to 13^+6^ weeks) could be used to predict the risk of short cervix at 20–24 weeks of gestation. We systematically evaluated seven ML algorithms and demonstrated the feasibility of early prediction. Among these, the XGBoost algorithm showed the most favorable performance and was selected for further analysis. This approach offers a clinically meaningful prediction window of approximately six weeks, enabling earlier risk stratification and timely intervention to reduce the likelihood of sPTB. Moreover, our model may serve as a new framework for interpreting cervical length assessments, offering insights into the early clinical indicators that precede cervical shortening and guiding more personalized prevention strategies in high-risk populations for sPTB.

## 2. Materials and Methods

### 2.1. Study Participants and Cervical Length Assessment

From January 2020 to December 2020, 1480 women with singleton pregnancies were recruited from Shanghai First Maternity and Infant Hospital, Tongji University School of Medicine. At enrollment (11^+0^ to 13^+6^ weeks gestation), cervical length assessment by TVUS was performed in a subset of participants based on clinical indications, with all measured cases showing normal cervical length (≥25 mm). For the remaining participants, cervical length was not assessed due to the absence of clinical indications.

All participants underwent standardized cervical length measurement by TVUS at 20–24 gestational weeks. In this study, a short cervix was defined as a cervical length < 25 mm at this timepoint [16]. Of these women, 1104 had a normal cervical length, and 376 had a short cervical length in mid-pregnancy. Women identified with a short cervix during mid-pregnancy were managed according to clinical guidelines, which included closer follow-up, vaginal progesterone administration, or cerclage placement, depending on individual risk factors and physician discretion.

The study plan was approved by the Ethics Committee of Shanghai First Maternity and Infant Hospital, Tongji University School of Medicine (approval number: KS22221).

The inclusion criteria were as follows: (1) singleton pregnant woman with at least one risk factor for sPTB (<37 weeks), such as a history of mid-pregnancy loss or preterm birth, a history of cervical surgery or electrocautery, a history of cervical cerclage, advanced age (defined as ≥35 years), or uterine malformation; (2) women who underwent regular prenatal follow-up and delivered at our hospital; and (3) Women who either had a normal cervical length at enrollment (based on TVUS in those with clinical indications for short cervix or preterm birth) or who did not undergo cervical length assessment at enrollment (due to the absence of these high-risk factors), and all underwent standardized TVUS monitoring of cervical length in mid-pregnancy (20–24 weeks). The exclusion criteria were as follows: (1) delivery before 24 weeks of gestation or iatrogenic preterm birth; (2) twin gestation, fetal chromosomal abnormalities or severe malformations, or selective fetal reduction after 14 weeks of gestation; and (3) loss to follow-up.

### 2.2. Data Processing and Cohort Division

Variables with missing values were examined prior to analysis. As the proportion of missing data was low (<5%), a complete case analysis was performed. This approach was chosen to preserve the integrity of the dataset without introducing imputation-related assumptions. We acknowledge that the exclusion of incomplete cases may introduce a degree of selection bias; however, given the low rate of missingness, the impact on model performance and generalizability is expected to be minimal.

All included pregnant women were randomly divided into training and testing cohorts at a 7:3 ratio using base R. The sample() function was applied to randomly assign individuals to each group, and a fixed random seed (set.seed(1000)) was used to ensure reproducibility of the data split. The training set was used for model development, while the testing set was reserved for performance evaluation.

### 2.3. Multimodal Data for Predicting Short Cervix in Mid-Pregnancy

The cervix was visualized in real time with an ultrasound probe placed in the vagina. Cervical length was measured as the distance between the internal os and the external os along the endocervical canal [17]. To ensure accuracy, the image was obtained with the entire cervical canal visible, the internal os clearly defined, and minimal pressure applied to avoid distortion. Three measurements were performed, and the shortest technically acceptable measurement was taken as the final result [16,18].

Multimodal data collected during the first trimester (11^+0^ to 13^+6^ weeks of gestation) were used to predict short cervix in mid-pregnancy (Table A1). Candidate predictors were selected based on prior literature and their biological plausibility in relation to cervical shortening and sPTB [19,20]. These clinical and laboratory parameters were chosen to capture both maternal background and biological markers, particularly those related to infection and inflammation, which are implicated in cervical remodeling and sPTB risk.

Clinical variables included maternal age, pre-pregnancy BMI, gravidity, parity, history of second-trimester pregnancy loss, previous preterm birth, mode of conception, uterine malformations (diagnosed by transvaginal 3D ultrasound or MRI [21]), and history of cervical surgery. These variables were included due to prior studies reporting associations with cervical insufficiency and preterm birth outcomes, even if the exact biological mechanisms remain unclear [22,23].

Laboratory variables consisted of vaginal microbiological testing and peripheral blood inflammatory markers. Vaginal secretion samples were collected between 11^+0^ and 13^+6^ weeks of gestation and analyzed for vulvovaginal candidiasis, trichomonad, mycoplasma, bacterial vaginosis, and vaginal microbiological culture (VMC). VMC was performed using standard aerobic techniques on Columbia blood agar and MacConkey agar plates. A result was considered positive if colony counts were ≥10^5^ CFU/mL [24]. Vulvovaginal candidiasis was diagnosed by 10% potassium hydroxide (KOH) smear microscopy, based on the identification of pseudohyphae or budding yeast [25]. Trichomonad was primarily detected by wet mount microscopy for visualization of motile trichomonads [26]. Mycoplasma species were identified using culture methods with commercial nucleic acid amplification tests (NAATs) [27]. Bacterial vaginosis was diagnosed by Gram-stained smear microscopy using Nugent scoring criteria [28]. Peripheral venous blood samples were also collected during the same gestational window to determine WBC, neutrophil percentage, and absolute neutrophil count using an automated hematology analyzer. Elevated leukocyte levels reflect systemic inflammatory status, which has been linked to intrauterine inflammation and subsequent cervical remodeling [5,29,30].

### 2.4. Predictive Variables

Feature selection involved automatically removing unnecessary features and selecting a subset of features to be used in predictive modeling. Tree models (Boruta), regularization techniques (LASSO regression, elastic net), and naive Bayesian recursive feature elimination (RFE) are commonly employed methods for feature selection. The Boruta algorithm was a wrapper algorithm based on random forest classification. It was iterated over all variables to determine if they had a higher Z score than the shadow variables. Lasso analysis was performed using L1 regularization, and the coefficients of unimportant regression variables were set to zero. Elastic net regression was used to extend the generalized linear model by adding regularization with a mixed L1/L2 loss function [31]. Moreover, RFE, an iterative greedy algorithm, was used to gradually discard the lowest ranked features until the optimal set of features was reached [32]. The intersection sets of the variants predicted by the methods mentioned above were selected the final predictor variables.

### 2.5. Derivation and Validation Data

To reduce the impact of the data imbalance, we augmented the data by using the Synthetic Minority Oversampling Technique (SMOTE) for Nominal and Continuous features (SMOTE-NC) in the training set. The SMOTE is a commonly used oversampling technique for dealing with imbalanced datasets [33]. The SMOTE-NC is a variant of the SMOTE, used not only for continuous variables but also for categorical variables.

### 2.6. Model Development and Validation

We employed seven popular ML algorithms: logistic regression (LR), linear discriminant analysis (LDA), K-nearest neighbors (KNN), support vector machine (SVM), decision tree (DT), random forest (RF), and extreme gradient boosting (XGBoost). Among all the models, the LR and LDA models were parametric models, while the others were nonparametric ML models. We performed tenfold cross-validation to determine the best parameters for the parametric models, selected suitable hyperparameters for the nonparametric models in the training set, and ultimately applied them to the test set.

In addition to the randomly divided test set mentioned above, we also validated the ML models by bootstrap resampling methods. All ML models were evaluated based on 1000 datasets generated by the stratified bootstrapping technique. The bootstrap samples were used as the training set, and the out-of-bag data were used as the test set.

The performance of the predictive model was evaluated based on the receiver operating characteristic (ROC) curve and the area under the curve (AUC) in the test set. In addition, the accuracy, precision, F1 score, sensitivity (Recall), and specificity were computed to evaluate model performance. The calibrations of the models were evaluated by using calibration curves. Brier scores were used to calibrate the models. A lower Brier score indicated a greater degree of calibration. Furthermore, we employed decision curve analysis (DCA) to assess the possible clinical impacts of all our models.

To further assess model generalizability, an independent dataset from the same institution but a different time period (January 2025) was used for validation.

### 2.7. Model Interpretation

To interpret the model predictions, Shapley additive explanations (SHAP) values were used. According to game theory, SHAP scores correlate feature importance with Shapley values. The formula for calculating SHAP values was as follows:

$$Shapley\;Value=\sum_{S\subseteq \left\{\;1,...,n\right\}/i}\frac{|S|!(n-|S|-1)!}{n!}\left[f(S\cup i)-f(S)\right]$$

In this formula, n is the total number of features, S is the subset of features, and is the function model [34].

SHAP values were used to estimate the contribution of each feature to the predictive model and quantify the association of each variable with the outcome of a single person in the cohort.

**Figure 1:**
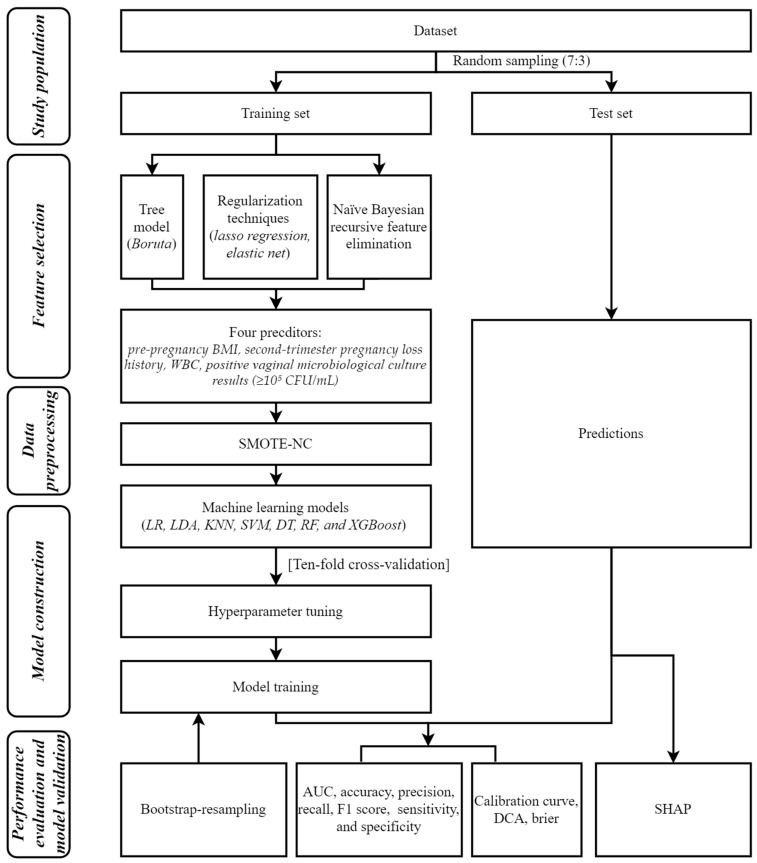
Flowchart of the study population, feature selection, data preprocessing, model construction, and performance evaluation.

The procedure for building the model for predicting short cervix in mid-pregnancy is shown in Figure 1.

### 2.8. Propensity Score Matching

To evaluate the independent effect of short cervix in mid-pregnancy on adverse pregnancy outcomes, propensity score matching (PSM) was adopted to match subjects in the short cervix and normal cervix groups by decreasing potential confounding bias. The matching ratio was 1:1, and the caliper value was 0.05.

### 2.9. Statistical Analysis

For continuous data, the Mann–Whitney U test was used for nonnormally distributed data, and Student’s *t* test was used for normally distributed data. Categorical data were compared using χ^2^ tests. In this study, all analyses were conducted using R statistical software (version 3.6.1, https://www.r-project.org/, accessed on 16 May 2025) and IBM SPSS Statistics 25. *p*-values less than 0.05 were considered to indicate statistical significance.

## 3. Results

### 3.1. Study Population Characteristics

The study cohort comprised 1480 pregnant women at high risk of sPTB. A total of 376 women were diagnosed with a short cervix, defined as a cervical length of <25 mm measured by TVUS between 20 and 24 weeks of gestation, while 1104 had a normal cervical length. The pregnant women with a short cervical length had a mean age of 33.8 ± 3.37 years, and those with a normal cervical length had a mean age of 33.9 ± 3.42 years. Significant differences were observed between the short and normal cervical length groups in several clinical and laboratory parameters, including pre-pregnancy BMI, pregnancy history, mode of conception, cervical surgery history, vaginal infections, and WBC and neutrophil counts. Age and history of first-trimester pregnancy loss did not differ significantly between groups. The clinical features of the included population are shown in Table 1.

### 3.2. Variable Screening

**Figure 2:**
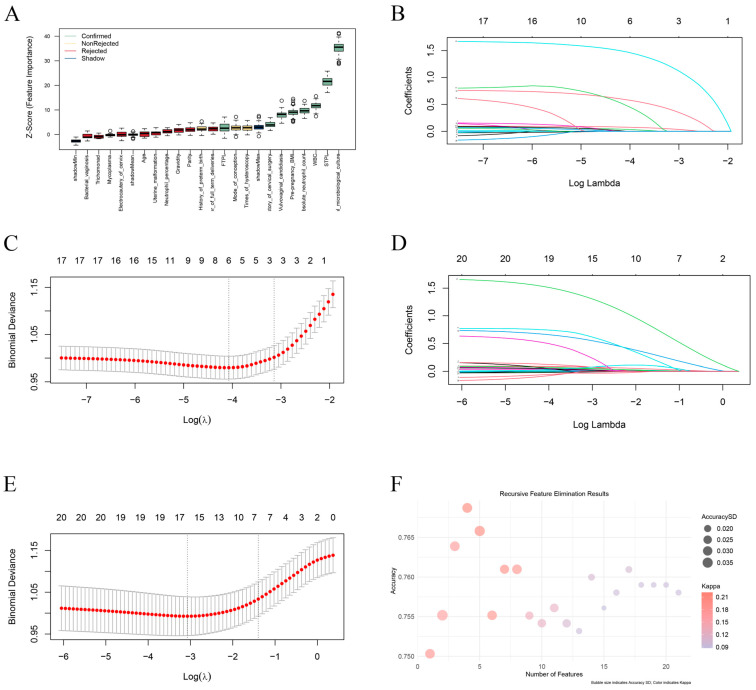
Tree model feature selection, regularization techniques and naive Bayesian recursive feature elimination. (**A**) Boruta selection of 21 features with importance rankings. Green is used for important variables, yellow is used for nonrejected variables, red is used for rejected variables, and blue is used for shadow variables. Error bars indicate the variability of importance scores across multiple iterations. *Y*-axis values represent the relative importance scores of each feature, which are dimensionless and do not have specific units. (**B**) Tenfold cross-validation of LASSO regression. (**C**) LASSO coefficient profiles of the 21 variables. The lower horizontal axis represents the value of log λ, the vertical axis represents the coefficient estimates, and the upper horizontal axis represents the number of variables with nonzero coefficients. (**D**) Tenfold cross-validation of the elastic net. (**E**) The elastic net regularized coefficient profiles of the 18 variables. (**F**) Bubble plot of the accuracy and number of features obtained by naive Bayesian recursive feature elimination.

**Figure 3:**
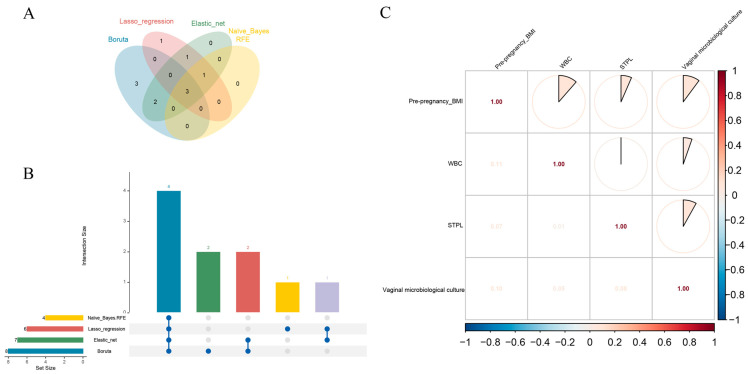
The intersection of the results of feature selection and collinearity analysis. (**A**) Venn diagram displaying features among the four selection techniques. (**B**) UpSet plot for features among the four selection techniques. (**C**) Heatmap of correlations between predictive factors. The colors represent positive (red) and negative (blue) correlations.

The full dataset was randomly divided into two exclusive datasets, with 70% comprising the training set (N = 1033) and 30% comprising the test set (N = 447). Four different feature selection methods were applied to select optimal predictive variables for short cervix in mid-pregnancy. We first applied the Boruta algorithm to 21 original features from the general information, medical history and laboratory examination aspects (Table A1) to eliminate nonrelevant features. Using the Boruta algorithm, 8 features were identified as important: pre-pregnancy BMI, history of first-trimester pregnancy loss, history of second-trimester pregnancy loss, history of cervical surgery, WBC, absolute neutrophil count, presence of vulvovaginal candidiasis, and positive vaginal microbiological culture results (VMC ≥ 10^5^ CFU/mL) measured between 11 + 0 and 13 + 6 weeks of gestation. These are presented in order of their Boruta importance in Figure 2A. Next, we separately performed feature selection using LASSO regression (Figure 2B,C) and elastic net algorithms (Figure 2D,E). Naive Bayesian RFE was also applied in tenfold cross-validation (Figure 2F). Taken together, the 4 studied predictors were significantly different for all the algorithms mentioned above. Pre-pregnancy BMI, a history of second-trimester pregnancy loss, WBC, and positive vaginal microbiological culture results (VMC ≥ 10^5^ CFU/mL) measured between 11 + 0 and 13 + 6 weeks of gestation (Figure 3A,B, Table A1). A correlation matrix was generated, and the Kendall tau correlation coefficients were displayed after hierarchical clustering as a heatmap. There was no high collinearity among the suggested variables (Figure 3C).

### 3.3. Data Preprocessing

In the training set, the proportion of women with short cervix in mid-pregnancy was significantly lower than that of healthy individuals (25.5% vs. 74.5%). In the testing set, there was a similar situation (25.3% vs. 74.7%). There was an important imbalance in the data described above (Table A2). Therefore, the SMOTE-NC was used.

### 3.4. Construction and Assessment of ML Models

We employed the four feature variables selected above to construct the prediction model. Seven machine learning models were constructed, including LR, LDA, KNN, SVM, DT, RF, and XGBoost models. Tenfold cross-validation was implemented on the training set to optimize the parameters. The optimal parameters or hyperparameters of all the models are presented in Table A3.

**Figure 4:**
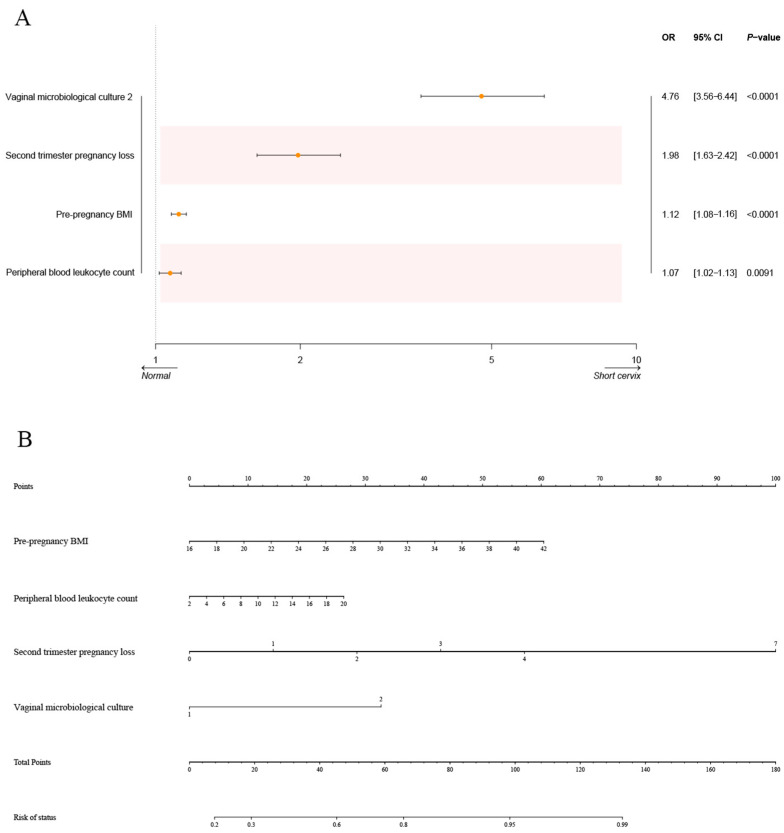
Model construction based on multivariate logistic regression analysis. (**A**) A forest plot showing the results of the multivariate logistic regression analysis. (**B**) Nomogram for predictive factors in the LR model. The value of each predictor is located on each variable axis, a line is drawn upward to determine the number of points for each variable value; the number of points for all variables is summed, and a line is drawn from the total number of points-axis to determine the probability of short cervix at the lower line.

**Figure 5:**
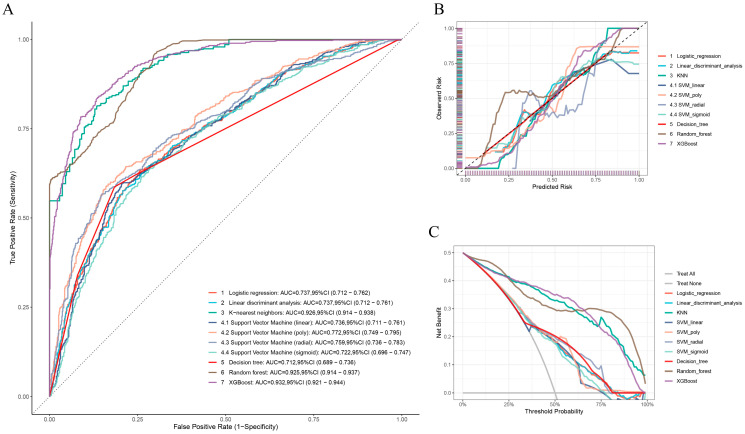
Evaluation of the model performance on the training set. (**A**) Evaluation of the seven machine learning models based on the AUC of the ROC curve in the training set. (**B**) Calibration curves of the seven machine learning models in the training set. (**C**) DCA curves of the seven machine learning models in the training set.

The LR model was applied to examine the four factors selected above (Figure 4A). Subsequently, the nomogram was constructed, as shown in Figure 4B. “Point” in the figure represents the individual scores and total scores corresponding to each variable at different values, and the last row is the risk of short cervix in mid-pregnancy. We also tested six other models. The SVM model includes four different algorithms: linear SVM, polynomial SVM, radial basis kernel (RBF)-SVM, and sigmoid SVM. The ROC curves of the training set based on all models are reported in Figure 5A. In the training set, the KNN (AUC = 0.926, 95% CI: 0.914–0.938), RF (AUC = 0.925, 95% CI: 0.914–0.937), and XGBoost (AUC = 0.932, 95% CI: 0.921–0.944) models had relatively high AUC values. The DeLong test was used to compare the AUCs among the three models in the training set, and there was no statistically significant difference in the AUCs. To further evaluate the utility of the models, calibration curves were generated (Figure 5B). The Brier score was used to evaluate the calibration of the model, and the XGBoost model exhibited high calibration (Table 2). In addition, to illustrate the clinical utility of the models, DCA was implemented. We concluded that the XGBoost model displayed greater net clinical benefits than the other models (Figure 5C). The models were also evaluated using six other performance measures: accuracy, precision, F1 score, sensitivity (Recall), and specificity (Table 2). The XGBoost model had the best accuracy (0.849) and F1 score (0.848) and relatively high sensitivity (0.838) and specificity (0.861) values. After comprehensive consideration, the XGBoost model was considered optimal.

### 3.5. Verification of the ML Models

**Figure A1:**
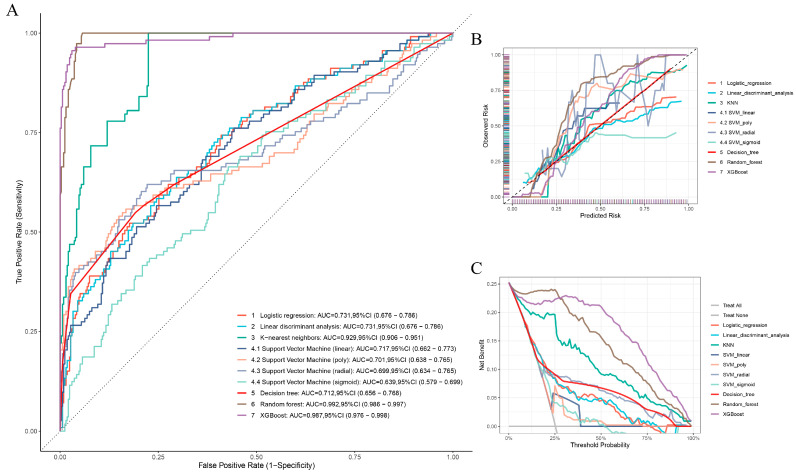
Evaluation of the model performance on the test set. (**A**) Evaluation of the seven machine learning models based on the AUC of the ROC curve in the test set. (**B**) Calibration curves of the seven machine learning models in the test set. (**C**) DCA curves of the seven machine learning models in the test set.

The models were validated using the testing set. In the testing set, the RF model had the highest AUC (0.992, 95% CI: 0.986–0.997), while the XGBoost model had the second highest AUC (0.987, 95% CI: 0.976–0.998) (Figure A1A). By comparing the AUCs of the RF model and XGBoost model in the test set, we found no significant differences between the best and second-best models (DeLong test, Z = 0.63986, *p* = 0.5223). In contrast, the XGBoost model, which had higher Recall (0.850) and F1 score (0. 910), was more suitable for clinical prediction. Additionally, we observed that the calibration curve of the XGBoost model was close to the ideal curve, suggesting that the model fit well (Figure A1B, Table A4). This model also had the greatest net benefit compared with the other models (Figure A1C). All the above results confirmed that the XGBoost model was the optimal model for use in first trimester to predict short cervix in mid-pregnancy. The results of the bootstrap validation can be found in Table A5 and Table A6. There were some similar results compared to the 7:3 dataset division.

**Figure A2:**
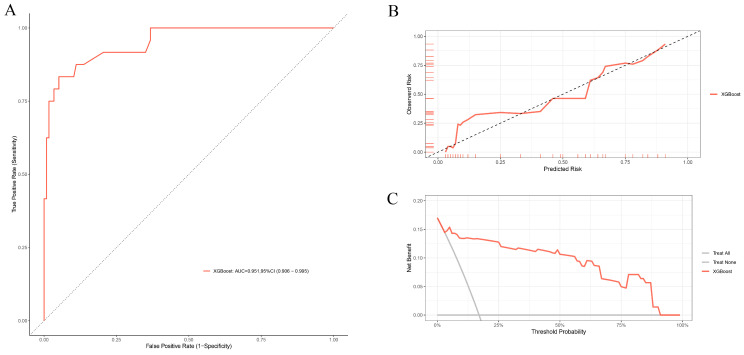
Evaluation of the model performance on an independent dataset collected in January 2025 from pregnant women during their registration at Shanghai First Maternity and Infant Hospital, Tongji University School of Medicine. (**A**) Evaluation of the XGboost model based on the AUC of the ROC curve. (**B**) Calibration curves of the XGboost model. (**C**) DCA curves of the XGboost model.

Additionally, we validated the model using an independent dataset collected in January 2025 from pregnant women during their registration at Shanghai First Maternity and Infant Hospital, Tongji University School of Medicine. The dataset comprised 141 patients, with basic information provided in Table A7. The XGBoost model exhibited excellent performance, achieving an AUC of 0.951 (95% CI: 0.906–0.995), a Recall of 0.708, and an F1 score of 0.791, further confirming its robust predictive capabilities (Figure A2).

### 3.6. Interpretability Analysis for the Optimal Model

**Figure 6:**
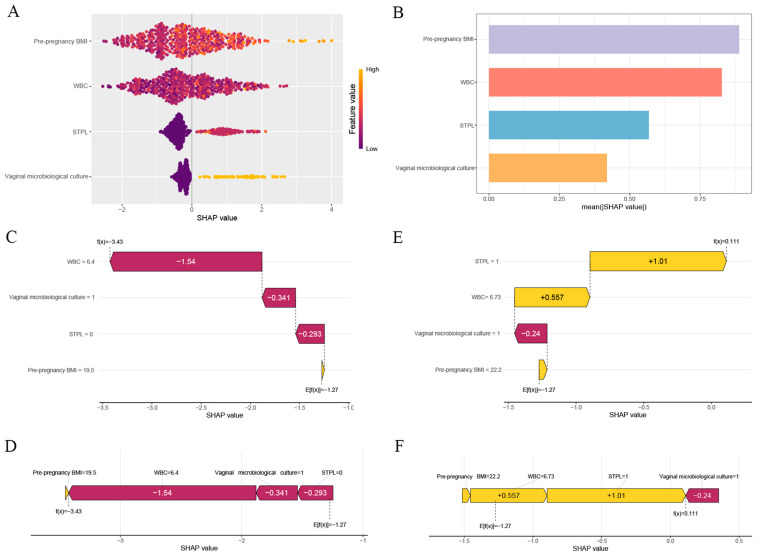
SHAP analysis results of the XGBoost model. (**A**) Scatter plots of the mean absolute SHAP value for each predictor. (**B**) Feature importance analysis using the SHAP method. (**C**,**E**) Waterfall plots showing the predicted risk of short cervix or normal cervix in two subjects. (**D**,**F**) Force plots showing the predicted risk of short cervix or normal cervix in two subjects.

We use the SHAP method to evaluate the interpretability of the XGBoost model. The scatter plots show the SHAP value of each feature for each sample (Figure 6A). All features were sorted in descending order of importance by the sum of the SHAP values of all the samples, and the SHAP values were used to show the distribution of the impact of each feature on the model (Figure 6B). The SHAP values were computed separately for the four variables (pre-pregnancy BMI = 0.889, WBC = 0.827, history of second-trimester pregnancy loss = 0.568, and positive vaginal microbiological culture results ≥10^5^ CFU/mL = 0.419). The waterfall plot and force plot visualized the prediction results for a single sample based on the model. Figure 6C,D shows the interactions of the risk factors for short cervix in mid-pregnancy in a 34-year-old woman. WBC of 6.4 and negative vaginal microbiological culture results (VMC < 10^5^ CFU/mL) were significant influencing factors for not having a short cervix in mid-pregnancy. A pregnant woman with a history of second-trimester miscarriage and the WBC equal to 6.73 before the 14th week of pregnancy was considered to have a high probability of having a short cervix in mid-pregnancy (Figure 6E,F).

### 3.7. sPTB Rate in the Normal and Short Cervix Groups

For pregnancy outcomes, in the group with a short cervix in mid-pregnancy, the sPTB (<37 weeks) rate was high at 41.2%, whereas the rate reached only 9.7% in the normal cervix group. Similarly, the sPTB rate (<34 weeks) was 31.6% in the short cervix group, while it was only 2.9% in the normal cervix group. The incidence of sPTB significantly differed between the two groups with different cervical lengths (Table 1). To reduce confounder effects, PSM was performed and included 21 baseline variables. After PSM, the differences in the baseline characteristics disappeared; however, the rates of sPTB (<34 weeks and <37 weeks) remained significantly different between the groups (Table A8). This demonstrated a close correlation between a short cervix in mid-pregnancy and sPTB.

## 4. Discussion

In this study, we aimed to address a clinically important challenge: the early identification of pregnant women at risk for a short cervix in mid-pregnancy, a key risk factor for sPTB. While TVUS at 20–24 weeks is the standard for detecting cervical shortening, it provides limited opportunity for early intervention. By leveraging routinely collected clinical and laboratory data from the first trimester (11 + 0 to 13 + 6 weeks), we applied and validated multiple ML models capable of predicting short cervix approximately six weeks in advance. This early prediction window holds substantial potential for improving pregnancy management by enabling timely preventive strategies, ultimately improving maternal and neonatal outcomes.

Our study highlights the importance of four key factors—pre-pregnancy BMI, history of second-trimester pregnancy loss, WBC, and positive vaginal microbiological culture (VMC ≥ 10^5^ CFU/mL, 11 + 0 to 13 + 6 weeks)—in predicting short cervix in mid-pregnancy. While these factors have previously been associated with preterm birth and cervical shortening, the role of these predictors during the first trimester has not been systematically explored. Previous studies have highlighted the association of high BMI and elevated leukocyte counts with increased risk of preterm birth, but these studies mainly focused on later stages of pregnancy, particularly the second and third trimesters [35,36,37]. Our study builds on this body of work by integrating these variables into a machine learning model applied to first-trimester data, which enables earlier risk identification and provides a more comprehensive risk assessment tool. This approach allows for earlier intervention, well before the usual 20–24-week screening window.

These factors were then used as input variables to apply seven different ML models. One of the key findings of our study was the relationship between pre-pregnancy BMI and short cervix. The relationship between pre-pregnancy BMI and short cervix remains controversial. Kaline Gomes Ferrari Marquart et al. [38] and Georgios Daskalakis [39] proposed that a high BMI reduces the risk of a short cervix in mid-pregnancy; however, Eleazar E Soto-Torres et al. suggested that a high BMI increases the risk [40]. In this study, a high pre-pregnancy BMI was considered a risk factor for a short cervix in mid-pregnancy. This variability in findings underscores the complexity of the relationship between BMI and pregnancy outcomes, which is likely influenced by multiple factors, including systemic inflammation, adipokine production, and mechanical forces on the cervix. Further research is needed to better understand the mechanisms by which BMI affects cervical length, especially during the early stages of pregnancy.

In addition to BMI, WBC was found to be an important predictor of short cervix, which is consistent with earlier research linking systemic inflammation to cervical remodeling. By studying serum markers, Simhan HN et al. showed that a shortened cervical length in mid-pregnancy may be associated with systemic inflammation [5]. The WBC is a marker of maternal inflammation and is associated with early sPTB in singleton pregnant women with a shortened cervix in mid-pregnancy, but there is no direct evidence to support its relationship with cervical length [41]. In our research, WBC was also one of the most important predictors of a short cervix in mid-pregnancy. However, the exact mechanism by which leukocyte levels influence cervical length is still unclear. Some studies propose that an inflammatory response triggered by infections or other systemic factors could contribute to cervical changes, but this hypothesis requires further validation.

Another novel aspect of our study is the identification of positive vaginal microbiological culture ≥10^5^ CFU/mL during the first trimester as a risk factor for short cervix. This finding is consistent with that of Van Lierde S et al. suggesting that vaginal infection in first trimester may directly participate in the process of cervical shortening [42]. While the exact pathway remains speculative, it is possible that infections during early pregnancy induce an inflammatory response, leading to the degradation of cervical tissue and subsequent shortening. The role of bacterial infections in cervical changes and preterm birth is a critical area for future research, particularly given the potential for early intervention with antibiotics or other treatments.

Furthermore, in our study, a history of mid-pregnancy loss was a strong predictor of a short cervix in mid-pregnancy. This was consistent with the findings of an earlier study [38]. A history of mid-pregnancy loss is also a strong predictor of sPTB [20,43], which is further reinforced by our data. In our study, PSM was applied to balance the baseline characteristics, and we found that the short cervix in mid-pregnancy group had a noticeably greater preterm birth rate than the normal cervix group. Women with a history of mid-pregnancy loss are at increased risk for short cervix and sPTB, suggesting that reproductive history is an important factor in predicting future pregnancy outcomes. This emphasizes the need to consider both obstetric history and early biomarkers in assessing the risk of preterm birth.

To establish the best performing model, we developed and compared several machine learning algorithms based on their predictive power using the selected input variables. The KNN, RF, and XGBoost models achieved high AUCs in the training set, but the differences were not statistically significant. However, the XGBoost model had a better calibration curve and DCA performance. It also had the best accuracy (0.849) and F1 score (0.848), and relatively high Recall (0.838). Therefore, the XGBoost model was the best ML model for the prediction of short cervix in mid-pregnancy among all seven ML models. Although the XGBoost (value = 0.987) model had the second highest AUC value and the RF model (value = 0.992) had the highest AUC value in the test set, these values were not significantly different. The high AUC observed in the test set can be attributed to the fact that we used SMOTE-NC to balance the training set, while the test set, reflecting a real-world distribution, was imbalanced. The test set contained a higher proportion of negative cases, which made the model more likely to predict the majority class (negative) accurately, resulting in higher specificity. In imbalanced datasets, even a model that predicts mostly the negative class can achieve high specificity, which may inflate the AUC. Therefore, while the AUC is high, it should be interpreted with caution, as it primarily reflects the model’s ability to predict the majority class, rather than its performance across all categories. The RF model, though achieving a high AUC, was significantly less sensitive than the XGBoost model, particularly due to its lower Recall, making it less suitable for predictive modeling in this context. The XGBoost model also performed the best according to bootstrap validation. Thus, we believe that the XGBoost model has better clinical applicability and is more effective in predicting short cervix in mid-pregnancy. To further assess the robustness and generalizability of the XGBoost model, we performed an additional validation using an independent dataset from our hospital, collected in January 2025. This validation supports the model’s reliability and potential applicability in clinical settings.

In the SHAP plot of the XGBoost model, we explained the feature contribution based on the SHAP value. These findings indicated that positive SHAP values were associated with a greater probability of short cervix in mid-pregnancy, while negative SHAP values were associated with a lower probability of short cervix in mid-pregnancy. In this research, pre-pregnancy BMI (0.889) was the most significant predictor, and the second most important predictor was the WBC (0.827). According to the available literature, a high pre-pregnancy BMI and an increased WBC in first trimester are also important risk factors for sPTB [29,44,45].

Nevertheless, we acknowledge several limitations that cannot be ignored. First, we classified cervical length as a binary variable (<25 mm vs. ≥25 mm) during the 20–24-week gestational period, which may have oversimplified the complex relationship between cervical shortening and the risk of sPTB. This simplification may have missed finer distinctions in risk, and we plan to refine this approach in future studies. Additionally, our study only assessed cervical length at a single timepoint (20–24 weeks), which may limit our ability to capture cases where cervical shortening occurs progressively. Multiple follow-up assessments would improve our ability to track changes over time. Furthermore, while we used an independent updated dataset for external validation, the absence of an external cohort from different hospitals or countries may limit the generalizability of our model. Our study was conducted at a single center with a predominantly Chinese population, which may not fully reflect the risk profiles of other ethnic groups. Moreover, our study population consisted of women with at least one risk factor for preterm birth, meaning it does not represent a low-risk population. Therefore, the findings may not be applicable to women at lower risk for preterm birth. Future multi-center, international studies are needed to validate the model across diverse populations. Future multi-center, international studies are needed to validate the model across diverse populations. Finally, while our model demonstrates promising predictive capabilities, it does not yet provide a specific clinical threshold for interventions. Determining such thresholds would require further investigation, integrating clinical decision-making criteria and expert consensus. Future research will aim to refine our model and explore the potential for actionable clinical guidelines that can inform timely interventions in at-risk pregnancies.

## 5. Conclusions

In conclusion, this study applied machine learning methods to predict the risk of short cervix in mid-pregnancy using first-trimester clinical data. Four key predictive factors for short mid-trimester cervical length included pre-pregnancy BMI, WBC, prior second-trimester pregnancy loss, and positive vaginal microbiological culture results (VMC ≥ 10^5^ CFU/mL). Among the models evaluated, the XGBoost model demonstrated the best performance in predicting short cervix. By offering an early prediction window of approximately six weeks before standard second-trimester screening, this model provides an opportunity for earlier intervention and better risk management. While the findings are based on a cohort with an elevated risk for preterm birth, they underscore the potential of machine learning as a valuable tool for enhancing early detection and prevention strategies for sPTB in high-risk clinical settings.

## Figures and Tables

**Table 1:** Baseline characteristics comparing the case group (short cervix) and control group (normal cervix).

	Predictive Variables	Short Cervix (N = 376)	Normal Cervix (N = 1104)	*p*-Values
**General information**	**Age**			
	Mean (SD)	33.8 (3.37)	33.9 (3.42)	0.484 ^a^
	**Pre-pregnancy BMI**			
	Mean (SD)	23.5 (3.61)	22.0 (3.14)	**<0.001 ^a^**
	**Gravidity**			
	Median [Min, Max]	3.00 [2.00, 9.00]	3.00 [2.00, 9.00]	**0.021 ^b^**
	**Parity**			
	Median [Min, Max]	0 [0, 3.00]	0 [0, 3.00]	0.623 ^b^
	**Number of full-term deliveries**			
	Median [Min, Max]	0 [0, 3.00]	0 [0, 3.00]	**0.005 ^b^**
**Medical history**	First trimester pregnancy loss (FTPL) *			
	Median [Min, Max]	1.00 [0, 8.00]	1.00 [0, 8.00]	0.17 ^b^
	Second trimester pregnancy loss (STPL) *			
	Median [Min, Max]	0 [0, 7.00]	0 [0, 4.00]	**<0.001 ^b^**
	**History of preterm birth**			
	Median [Min, Max]	0 [0, 2.00]	0 [0, 1.00]	**0.015 ^b^**
	**Mode of conception**			
	Natural conception	230 (61.2%)	732 (66.3%)	**<0.001 ^c^**
	Ovulation induction	26 (6.9%)	27 (2.4%)	
	IVF-ET	8 (2.1%)	9 (0.8%)	
	ICSI	84 (22.3%)	218 (19.7%)	
	PGD	28 (7.4%)	118 (10.7%)	
	**Uterine malformation**			
	Normal uterus	369 (98.1%)	1089 (98.6%)	0.148 ^c^
	Bicornuate uterus	0 (0%)	5 (0.5%)	
	Septate uterus	7 (1.9%)	10 (0.9%)	
	**History of cervical surgery**			
	None	963 (87.2%)	334 (88.8%)	**0.015 ^c^**
	Cervical LEEP cone resection	109 (9.9%)	23 (6.1%)	
	Cervical conization	32 (2.9%)	19 (5.1%)	
	**Times of hysteroscopy**			
	Mean (SD)	0.601 (1.22)	0.544 (0.988)	0.415 ^b^
	Median [Min, Max]	0 [0, 9.00]	0 [0, 9.00]	
	**Electrocautery of cervix**			
	No	1098 (99.5%)	375 (99.7%)	0.686 ^c^
	Yes	6 (0.5%)	1 (0.3%)	
**Laboratory examination**	**Vulvovaginal candidiasis**			
	No	305 (81.1%)	1016 (92.0%)	**<0.001 ^c^**
	Yes	71 (18.9%)	88 (8.0%)	
	**Trichomonad**			
	No	375 (99.7%)	1103 (99.9%)	0.444 ^c^
	Yes	1 (0.3%)	1 (0.1%)	
	**Mycoplasma**			
	No	276 (73.4%)	866 (78.4%)	**0.047 ^c^**
	Yes	100 (26.6%)	238 (21.6%)	
	**Bacterial vaginosis**			
	No	336 (89.4%)	1010 (91.5%)	0.213 ^c^
	Yes	40 (10.6%)	94 (8.5%)	
	**Vaginal microbiological culture (** **≥10^5^** **CFU/mL)**			
	No	242 (64.4%)	1002 (90.8%)	**<0.001 ^c^**
	Yes	134 (35.6%)	102 (9.2%)	
	**WBC**			
	Mean (SD)	9.15 (2.38)	8.60 (2.15)	**<0.001 ^a^**
	**Neutrophil percentage**			
	Mean (SD)	72.3 (6.60)	71.8 (6.67)	0.258 ^a^
	**Absolute neutrophil count**			
	Mean (SD)	6.67 (2.07)	6.23 (1.85)	**<0.001 ^a^**
**Secondary outcome**	**sPTB (<34 weeks)**			
	No	257 (68.4%)	1072 (97.1%)	**<0.001 ^c^**
	Yes	119 (31.6%)	32 (2.9%)	
	**sPTB (<37 weeks)**			
	No	221 (58.8%)	997 (90.3%)	**<0.001 ^c^**
	Yes	155 (41.2%)	107 (9.7%)	

* First trimester pregnancy loss includes both spontaneous abortion and elective abortion; Second trimester pregnancy loss includes both spontaneous abortion and elective abortion. a. Student’s *t* test. b. Mann–Whitney U test. c. Pearson’s chi-squared (χ^2^) test.

**Table 2:** Performance of the machine learning models (training set).

Model	Accuracy	Precision	F1 Score	Sensitivity (Recall)	Specificity	Brier
Logistic regression	0.6831169	0.7288961	0.6479076	0.5831169	0.7831169	0.2086745
LDA	0.6818182	0.708559	0.6391753	0.5636364	0.8	0.2090479
KNN	0.8272727	0.8173804	0.8299233	0.8428571	0.8116883	0.1185919
Linear SVM	0.6831169	0.7085799	0.6625173	0.6220779	0.7441558	0.2184744
Polynomial SVM	0.6798701	0.6520307	0.7067222	0.7714286	0.5883117	0.2038918
RBF-SVM	0.696104	0.732308	0.670423	0.618182	0.774026	0.2037133
Sigmoid SVM	0.672727	0.685237	0.66129	0.638961	0.706494	0.215311
DT	0.701299	0.760943	0.662757	0.587013	0.815584	0.2057295
RF	0.792208	0.901786	0.759399	0.655844	0.928571	0.1248432
XGBoost	0.849351	0.857713	0.847569	0.837662	0.861039	0.1153128

## Data Availability

Data are contained within the article or available upon request from the corresponding authors.

## Appendix A

**Table A1:** Descriptive Statistics of the Study Variables.

Predictors	Types	Values
Age	Discrete	/
**Pre-pregnancy BMI**	**Continuous**	**BMI = weight/height^2^ (kg/m^2^)**
Gravidity	Discrete	Number of pregnancies (including the current pregnancy)
Parity	Discrete	Number of births (excluding the current pregnancy)
Number of full-term deliveries	Discrete	Times of delivery without pregnancy loss or preterm birth (excluding the current pregnancy)
First-trimester pregnancy loss	Discrete	Times of miscarriages in the first trimester (excluding the current pregnancy)
**Second-trimester pregnancy loss**	**Discrete**	**Times of miscarriages in the second trimester (excluding the current pregnancy)**
History of preterm birth	Discrete	Times of preterm birth (excluding the current pregnancy)
Mode of conception	Categorical	Mode of conception for this pregnancy 1- Natural conception 2- Ovulation induction 3- IVF-ET 4- ICSI 5- PGD
Uterine malformation	Categorical	1- Normal uterus 2- Bicornuate uterus 3- Septate uterus
History of cervical surgery	Categorical	1- None 2- Cervical LEEP cone resection 3- Cervical conization
Times of hysteroscopy	Discrete	Number of hysteroscopic examinations
Electrocautery of cervix	Categorical	1- No 2- Yes
Vulvovaginal candidiasis	Categorical	Vulvovaginal candidiasis detection (tested between 11^+0^ and 13^+6^ weeks of gestation) 1- No 2- Yes
Trichomonad	Categorical	Trichomonad detection in vaginal secretions (tested between 11^+0^ and 13^+6^ weeks of gestation) 1- No 2- Yes
Mycoplasma	Categorical	Mycoplasma detection in vaginal secretions (tested between 11^+0^ and 13^+6^ weeks of gestation) 1- No 2- Yes
Bacterial vaginosis	Categorical	Bacterial vaginosis detection (tested between 11^+0^ and 13^+6^ weeks of gestation) 1- No 2- Yes
**Vaginal microbiological culture (** **≥10^5^** **CFU/mL)**	**Categorical**	**Bacterial culture of vaginal secretions (tested between 11 + 0 and 13 + 6 weeks of gestation)** **1- No** **2- Yes**
**WBC**	**Continuous**	**Absolute peripheral blood leukocyte count (tested between 11 + 0 and 13 + 6 weeks of gestation)**
Neutrophil percentage (%)	Continuous	Percentage of neutrophils in peripheral blood (tested between 11^+0^ and 13^+6^ weeks of gestation))
Absolute neutrophil count	Continuous	Absolute peripheral blood neutrophil values (tested between 11^+0^ and 13^+6^ weeks of gestation)

Note: Variables incorporated in machine learning models are shown in bold.

**Table A2:** Comparison of clinical features between the training and test groups.

	Predictive Variables	Trainset (N = 1033)	Test Set (N = 447)	*p*-Values
**General information**	**Age**			
	Mean (SD)	33.9 (3.42)	34.0 (3.38)	0.431 ^a^
	**Pre-pregnancy BMI**			
	Mean (SD)	22.4 (3.35)	22.3 (3.28)	0.797 ^a^
	**Gravidity**			
	Median [Min, Max]	3.00 [2.00, 9.00]	3.00 [2.00, 9.00]	0.296 ^b^
	**Parity**			
	Median [Min, Max]	0 [0, 3.00]	0 [0, 3.00]	0.128 ^b^
	**Number of full-term deliveries**			
	Median [Min, Max]	0 [0, 3.00]	0 [0, 3.00]	0.014 ^b^
**Medical history**	**First trimester pregnancy loss ***			
	Median [Min, Max]	1.00 [0, 8.00]	1.00 [0, 8.00]	0.025 ^b^
	**Second trimester pregnancy loss ***			
	Median [Min, Max]	0 [0, 7.00]	0 [0, 3.00]	0.628 ^b^
	**History of preterm birth**			
	Median [Min, Max]	0 [0, 2.00]	0 [0, 2.00]	0.379 ^b^
	**Mode of conception**			
	Natural conception	660 (63.9%)	302 (67.6%)	0.408 ^c^
	Ovulation induction	39 (3.8%)	14 (3.1%)	
	IVF-ET	14 (1.4%)	3 (0.7%)	
	ICSI	221 (21.4%)	81 (18.1%)	
	PGD	99 (9.6%)	47 (10.5%)	
	**Uterine malformation**			
	Normal uterus	1018 (98.5%)	440 (98.4%)	0.859 ^c^
	Bicornuate uterus	4 (0.4%)	1 (0.2%)	
	Septate uterus	11 (1.1%)	6 (1.3%)	
	**History of cervical surgery**			
	None	904 (87.5%)	393 (87.9%)	0.934 ^c^
	Cervical LEEP cone resection	92 (8.9%)	40 (8.9%)	
	Cervical conization	37 (3.6%)	14 (3.1%)	
	**Times of hysteroscopy**			
	Median [Min, Max]	0 [0, 9.00]	0 [0, 9.00]	0.137 ^b^
	**Electrocautery of cervix**			
	No	1026 (99.3%)	447 (100%)	0.11 ^c^
	Yes	7 (0.7%)	0 (0%)	
**Laboratory examination**	**Vulvovaginal candidiasis**			
	No	925 (89.5%)	396 (88.6%)	0.584 ^c^
	Yes	108 (10.5%)	51 (11.4%)	
	**Trichomonad**			
	No	1031 (99.8%)	447 (100%)	1 ^c^
	Yes	2 (0.2%)	0 (0%)	
	**Mycoplasma**			
	No	806 (78.0%)	336 (75.2%)	0.252 ^c^
	Yes	227 (22.0%)	111 (24.8%)	
	**Bacterial vaginosis**			
	No	936 (90.6%)	410 (91.7%)	0.554 ^c^
	Yes	97 (9.4%)	37 (8.3%)	
	**Vaginal microbiological culture (** **≥10^5^** **CFU/mL)**			
	No	865 (83.7%)	379 (84.8%)	0.643 ^c^
	Yes	168 (16.3%)	68 (15.2%)	
	**WBC**			
	Mean (SD)	8.73 (2.21)	8.75 (2.27)	0.855 ^a^
	**Neutrophil percentage**			
	Mean (SD)	72.0 (6.58)	71.7 (6.83)	0.359 ^a^
	**Absolute neutrophil count**			
	Mean (SD)	6.34 (1.88)	6.34 (1.99)	0.982 ^a^
**Outcome**	**Short cervix**			
	No	770 (74.5%)	334 (74.7%)	1 ^c^
	Yes	263 (25.5%)	113 (25.3%)	

* First trimester pregnancy loss includes both spontaneous abortion and elective abortion. Second trimester pregnancy loss includes both spontaneous abortion and elective abortion. a. Student’s *t* test. b. Mann–Whitney U test. c. Pearson’s chi-squared test.

**Table A3:** The optimum values of the parameters and hyperparameters for the ML models.

Models	Parameters/Hyperparameters	Optimum Value
LR	Coefficients	Pre-pregnancy BMI = 0.11290, peripheral blood leukocyte = 0.07022, second trimester pregnancy loss = 0.68365, vaginal microbiological culture 2 = 1.54878
LDA	Coefficients of linear discriminants	Pre-pregnancy BMI = 0.13325144, Peripheral blood leukocyte = 0.07801393, second trimester pregnancy loss = 0.73637384, vaginal microbiological culture 2 = 1.79646255
KNN	k, kernel	k = 9, kernel = “optimal”
Linear SVM	cost	cost = 0.1
Polynomial SVM	cost, degree, coef.0	cost = 1, degree = 5, coef.0 = 2
RBF-SVM	cost	cost = 1
Sigmoid SVM	cost, coef.0	cost = 0.1, coef.0 = 0
DT	cp	cp = 0.01038961
RF	mtry, ntree	mtry = 2, ntree = 400
XGBoost	nrounds, max_depth, eta = 0.3, gamma, colsample_bytree, min_child_weight, subsample	nrounds = 100, max depth = 3, eta = 0.4, gamma = 0, colsample bytree = 1, min child weight = 1, subsample = 1

**Table A4:** Performance of the machine learning models (test set).

Model	Accuracy	Precision	F1 Score	Sensitivity (Recall)	Specificity	Brier
Logistic regression	0.7919463	0.7173913	0.4150943	0.2920354	0.9610778	0.1587173
LDA	0.7964206	0.7115385	0.4484848	0.3274336	0.9550898	0.1593799
KNN	0.8389262	0.8153846	0.5955056	0.4690265	0.9640719	0.1025336
Linear SVM	0.7606264	0.875	0.1157025	0.0619469	0.997006	0.168591
Polynomial SVM	0.7941834	0.8387097	0.3611111	0.2300885	0.9850299	0.1620557
RBF-SVM	0.8098434	0.8333333	0.4516129	0.3097345	0.9790419	0.1500501
Sigmoid SVM	0.7472036	0.5	0.1102362	0.0619469	0.9790419	0.1836746
DT	0.8143177	0.8125	0.484472	0.3451327	0.9730539	0.1477355
RF	0.9038031	1	0.7650273	0.619469	1	0.06315483
XGBoost	0.9574944	0.9795918	0.9099526	0.8495575	0.994012	0.04911613

**Table A5:** Performance of the machine learning models according to the bootstrap method (training set).

Model	AUC	Accuracy	Precision	F1 Score	Sensitivity (Recall)	Specificity	Brier
Logistic regression	0.752 (0.711–0.794)	0.7901726	0.6585366	0.406015	0.2934783	0.9507909	0.1539881
LDA	0.751 (0.71–0.793)	0.7848606	0.6078431	0.4335664	0.3369565	0.9297012	0.1550962
KNN	0.935 (0.919–0.951)	0.8472776	0.785124	0.6229508	0.5163043	0.9543058	0.09834036
Linear SVM	0.74 (0.697–0.782)	0.7715803	0.5555556	0.4109589	0.326087	0.9156415	0.1685743
Polynomial SVM	0.708 (0.658–0.758)	0.7901726	0.7954545	0.3070175	0.1902174	0.9841828	0.1848635
RBF-SVM	0.732 (0.684–0.781)	0.8061089	0.75	0.4384615	0.3097826	0.9666081	0.1505253
Sigmoid SVM	0.68 (0.635–0.725)	0.752988	0.375	0.03125	0.01630435	0.9912127	0.1718315
DT	0.739 (0.697–0.78)	0.8180611	0.742268	0.5124555	0.3913043	0.9560633	0.1416904
RF	0.98 (0.973–0.987)	0.8937583	0.9814815	0.7260274	0.576087	0.9964851	0.07299181
XGBoost	0.971 (0.96–0.983)	0.9216467	0.9432624	0.8184615	0.7228261	0.9859402	0.06609167

**Table A6:** Performance of the machine learning models according to the bootstrap method (test set).

Model	AUC	Accuracy	Precision	F1 Score	Sensitivity (Recall)	Specificity	Brier
Logistic regression	0.757 (0.716–0.798)	0.7757909	0.6380952	0.4511785	0.3489583	0.928972	0.1592825
LDA	0.757 (0.716–0.798)	0.7675378	0.592	0.466877	0.3854167	0.9046729	0.160957
KNN	0.928 (0.91–0.945)	0.8363136	0.792	0.6246057	0.515625	0.9514019	0.1050932
Linear SVM	0.756 (0.715–0.797)	0.7634113	0.578125	0.4625	0.3854167	0.8990654	0.1692026
Polynomial SVM	0.706 (0.657–0.755)	0.7647868	0.8888889	0.2191781	0.125	0.9943925	0.1751815
RBF-SVM	0.735 (0.687–0.782)	0.786795	0.7466667	0.4194757	0.2916667	0.964486	0.1577546
Sigmoid SVM	0.702 (0.658–0.747)	0.7551582	0.5777778	0.3687943	0.2708333	0.928972	0.1740828
DT	0.75 (0.708–0.792)	0.8115543	0.8115543	0.8115543	0.8115543	0.8115543	0.145428
RF	0.981 (0.974–0.988)	0.9037139	1	0.7770701	0.6354167	1	0.07283245
XGBoost	0.972 (0.961–0.982)	0.9202201	0.9294872	0.8333333	0.7552083	0.9794393	0.07035735

**Table A7:** Clinical features of patients based on an independent dataset collected in January 2025 from pregnant women during their registration at Shanghai First Maternity and Infant Hospital, Tongji University School of Medicine.

	Predictive Variables	Short Cervix (N = 24)	Normal Cervix (N = 117)	*p*-Values
**General information**	**Age**			
	Mean (SD)	36.6(3.05)	38.6 (2.00)	0.018 ^a^
	**Pre-pregnancy BMI**			
	Mean (SD)	22.4 (2.89)	21.98 (2.89)	<0.001 ^a^
	**Gravidity**			
	Median [Min, Max]	2.00 [1.00, 9.00]	2.00 [1.00, 9.00]	<0.001 ^b^
	**Parity**			
	Median [Min, Max]	0 [0, 2.00]	0 [0, 3.00]	0.002 ^b^
**Medical history**	**Second trimester pregnancy loss ***			
	Median [Min, Max]	0.00 [0, 1.00]	0.00 [0, 1.00]	<0.001^b^
**Laboratory examination**	**Vaginal microbiological culture (** **≥10^5^** **CFU/mL)**			
	No	5 (20.8%)	112 (95.7%)	<0.001 ^c^
	Yes	19 (79.2%)	5 (4.3%)	
	**WBC**			
	Mean (SD)	10.17(2.56)	8.08 (1.45)	0.002 ^a^

* Second trimester pregnancy loss includes both spontaneous abortion and elective abortion. a. Student’s *t* test. b. Mann–Whitney U test. c. Pearson’s chi-squared test.

**Table A8:** Clinical features of patients in the short cervix and normal cervix groups after PSM.

	Predictive Variables	Short Cervix (N = 308)	Normal Cervix (N = 308)	*p*-Values
**General information**	**Age**			
	Mean (SD)	33.9 (3.40)	33.8 (3.26)	0.762 ^a^
	**Pre-pregnancy BMI**			
	Mean (SD)	23.1 (3.38)	23.2 (3.56)	0.575 ^a^
	**Gravidity**			
	Median [Min, Max]	3.00 [2.00, 9.00]	3.00 [2.00, 8.00]	0.602 ^b^
	**Parity**			
	Median [Min, Max]	0 [0, 3.00]	0 [0, 3.00]	0.884 ^b^
	**Number of full-term deliveries**			
	Median [Min, Max]	0 [0, 3.00]	0 [0, 3.00]	0.842 ^b^
**Medical history**	**First trimester pregnancy loss ***			
	Median [Min, Max]	1.00 [0, 8.00]	1.00 [0, 6.00]	0.821 ^b^
	**Second trimester pregnancy loss ***			
	Median [Min, Max]	0 [0, 3.00]	0 [0, 4.00]	0.642 ^b^
	**History of preterm birth**			
	Median [Min, Max]	0 [0, 2.00]	0 [0, 1.00]	0.883 ^b^
	**Mode of conception**			
	Natural conception	191 (62.0%)	197 (64.0%)	0.718 ^c^
	Ovulation induction	18 (5.8%)	17 (5.5%)	
	IVF-ET	4 (1.3%)	6 (1.9%)	
	ICSI	71 (23.1%)	59 (19.2%)	
	PGD	24 (7.8%)	29 (9.4%)	
	**Uterine malformation**			
	Normal uterus	303 (98.4%)	303 (98.4%)	1 ^c^
	Bicornuate uterus	0 (0%)	0 (0%)	
	Septate uterus	5 (1.6%)	5 (1.6%)	
	**History of cervical surgery**			
	None	275 (89.3%)	281 (91.2%)	0.729 ^c^
	Cervical LEEP cone resection	20 (6.5%)	17 (5.5%)	
	Cervical conization	13 (4.2%)	10 (3.2%)	
	**Times of hysteroscopy**			
	Median [Min, Max]	0 [0, 9.00]	0 [0, 9.00]	0.735 ^b^
	**Electrocautery of cervix**			
	No	307 (99.7%)	308 (100%)	1 ^c^
	Yes	1 (0.3%)	0 (0%)	
**Laboratory examination**	**Vulvovaginal candidiasis**			
	No	262 (85.1%)	259 (84.1%)	0.824 ^c^
	Yes	46 (14.9%)	49 (15.9%)	
	**Trichomonad**			
	No	307 (99.7%)	308 (100%)	1 ^c^
	Yes	1 (0.3%)	0 (0%)	
	**Mycoplasma**			
	No	233 (75.6%)	233 (75.6%)	1 ^c^
	Yes	75 (24.4%)	75 (24.4%)	
	**Bacterial vaginosis**			
	No	279 (90.6%)	272 (88.3%)	0.432 ^c^
	Yes	29 (9.4%)	36 (11.7%)	
	**Vaginal microbiological culture (** **≥10^5^** **CFU/mL)**			
	No	224 (72.7%)	229 (74.4%)	0.715 ^c^
	Yes	84 (27.3%)	79 (25.6%)	
	**WBC**			
	Mean (SD)	8.98 (2.33)	8.94 (2.38)	0.836 ^a^
	**Neutrophil percentage**			
	Mean (SD)	72.1 (6.64)	72.1 (7.01)	0.981 ^a^
	**Absolute neutrophil count**			
	Mean (SD)	6.54 (2.03)	6.51 (2.05)	0.884 ^a^
**Secondary outcome**	**sPTB (<34 weeks)**			
	No	221 (71.8%)	295 (95.8%)	<0.001^c^
	Yes	87 (28.2%)	13(4.2%)	
	**sPTB (<37 weeks)**			
	No	191 (62.0%)	273 (88.6%)	<0.001^c^
	Yes	117 (38.0%)	35 (11.4%)	

* First trimester pregnancy loss includes both spontaneous abortion and elective abortion. Second trimester pregnancy loss includes both spontaneous abortion and elective abortion. a. Student’s *t* test. b. Mann–Whitney U test. c. Pearson’s chi-squared test.
